# Knowledge, attitude, practices and associated factors of family planning among women living with hiv at the university of Gondar specialized hospital: a cross sectional study

**DOI:** 10.1186/s12905-024-03036-9

**Published:** 2024-04-12

**Authors:** Wudneh Simegn, Eman Hussen, Yossef Maru, Abdulwase Mohammed Seid, Liknaw Workie Limenh, Wondim Ayenew, Mihret Melese, Berhanemeskel Weldegerima Atsbeha

**Affiliations:** 1 Department of Social and Administrative Pharmacy, School of Pharmacy, College of Medicine and Health Sciences, University of Gondar, Gondar, Ethiopia; 2 Department of Clinical Pharmacy, School of Pharmacy,College of Medicine and Health Sciences, , University of Gondar, Gondar, Ethiopia; 3 Department of pharmaceutics, School of Pharmacy, College of Medicine and Health Sciences, , University of Gondar, Gondar, Ethiopia; 4 Department of Human Physiology, School of Medicine, College of Medicine and Health Science, University of Gondar, Gondar, Ethiopia

**Keywords:** Family planning, HIV positive women, Knowledge, Attitude, Practice

## Abstract

**Introduction:**

HIV/AIDS poses a significant health challenge in sub-Saharan African countries, with a disproportionate impact on women of reproductive age. The disparities in knowledge, attitudes, and practices related to family planning among women living with HIV can be intricate and multi-faceted. This study aimed to assess the knowledge, attitude, practice, and associated factors regarding family planning among the women living with HIV at the University of Gondar specialized hospital, Gondar, Ethiopia.

**Method:**

A cross-sectional study was carried out at the University of Gondar Teaching Referral Hospital, focusing on HIV-positive women of reproductive age who visited the ART unit from July 8–28, 2022. Data collection involved the use of pre-tested, structured questionnaires administered through interviews. The gathered data were entered into the electronic Kobo Collect platform and subsequently exported for analysis using SPSS version 26. Descriptive summaries, including frequencies, means, and percentages, were presented through tables and figures. Logistic regression was employed to identify potential predictors, presenting adjusted odds ratios with a 95% confidence interval and a significance level set at a *P*-value of 0.05.

**Results:**

A total of 328 study participants were included. About 93% of the study population had good knowledge about modern contraceptives, and about 94% of the study population had good knowledge about safer conception. Only 30.2% of the study population had knowledge of the dual contraceptive method. The attitude and practice of women towards family planning (FP) were 71.0% and 55.8%, respectively. The study revealed that the most commonly employed contraceptive method was injectable contraceptives, constituting 34.2% of usage. Having one and a greater number of children (AOR = 2.25, 95% CI: 1.10, 4.49), having discussions on fertility plans with healthcare providers (AOR = 2.20, 95% CI: 1.02, 4.761), and having good family planning practices (AOR = 2.15, 95% CI: 1.19, 3.87) were significantly associated with the attitude toward family planning. Married women (AOR = 1.88, 95% CI = 1.11, 3.1), able to read and write (AOR = 2.12, 95% CI:1.04,4.32), college and above educational level (AOR = 4.51, 95% CI:1.93,10.87), had discussion on fertility plan with healthcare providers (AOR = 5.09, 95% CI: 1.96, 13.24), knowledge about dual method (AOR = 1.95, CI: 1.08, 3.50), and knowledge about modern contraceptive methods (AOR = 7.24, 95% CI: 1.56, 33.58) were significantly associated with good practice of family planning.

**Conclusion:**

Women living with HIV exhibited notably high levels of knowledge regarding modern contraceptive methods and safer conception. The knowledge of the dual method was low. More than half of the study population had good practice in family planning. More than two-thirds of HIV-positive reproductive-age women had a good attitude about family planning. Having one or a greater number of children, having a discussion on a fertility plan with a healthcare provider, and having a good practice of family planning were significantly associated with a good attitude toward family planning. Married women, education status, discussions on fertility plans with healthcare providers, knowledge about dual methods, and knowledge about modern contraceptive methods were significantly associated with good family planning practices. The stakeholders should design interventions based on the aforementioned factors to improve the attitude and practice of family planning.

## Introduction

The human immunodeficiency virus/acquired immune deficiency syndrome (HIV/AIDS) epidemic is the most serious global public health problem, more particularly seen in low and middle-income countries [1]. Infection with HIV can be acquired mostly through unprotected sexual intercourse (both anal and vaginal), injectable drug use, receipt of tainted blood products, and mother-to-infant transmission (both perinatal infection and postpartum through breastfeeding) [2].

Women continue to bear a disproportionate impact of the HIV epidemic, especially in sub-Saharan Africa, where they constitute 58% of adults living with HIV [3]. This indicated that the prevention of HIV infection in women is, of course, required to prevent new infections in infants [4]. The risk of mother-to-child transmission is approximately 25% in the absence of ART, including breast-feeding, which accounted for approximately 5–10% in the first 6 months [5].

Family planning (FP) is defined as a way of thinking and living that is adopted voluntary upon the basis of knowledge, attitude, and responsible decisions by individuals and couples [6, 7]. It is a conscious effort by a couple to limit or space the number of children they have through the use of contraceptive methods [8, 9]. Family planning deals with the reproductive health of the mother, having adequate birth spacing, avoiding unwanted pregnancies and abortions, preventing sexually transmitted diseases, and improving the quality of life of the mother, fetus, and family as a whole [10]. Safer conception strategies (SCS) for HIV-affected couples, such as pre-exposure prophylaxis (PrEP) for HIV prevention in serodiscordant couples and antiretroviral therapy (ART) treatment as prevention (TasP), as well as timed unprotected intercourse, have made it possible for women to conceive with significantly less risk of HIV infection or transmission [10–12]. This is more obedient when women have knowledge, attitude, and practice about modern family planning methods for spacing and limiting [13]. Due to the greatly reduced risk of HIV transmission when using SCS and PMTCT strategies, along with the high fertility intentions and HIV burden among women of reproductive age, a reproductive rights approach toward managing the reproductive health of people with HIV (PHIV) is important [14–16]. This is accomplished through the dual application of methods, such as employing condoms to reduce the risk of HIV transmission and simultaneously utilizing at least one highly effective contraceptive method to avoid unintended pregnancies. The highly effective methods included oral contraceptive pills, injections, implants, IUDs, tubal ligation, vasectomy, or infertility [10].

HIV continues to be a major global public health issue, having claimed 38.4 million [33.9 million–43.8 million] people were living with HIV in 2021 and 1.5 million [1.1 million–2.0 million] people became newly infected in 2021. From this, 36.7 million [36.7 million–41.9 million] adults (15 years of age or older) and 1.7 million [1.3 million–2.1 million] children (0–14) were living with HIV, and 54% of all people living with HIV were women and girls [17]. In Africa, the most affected region, 25.7 million people live with HIV, and the region also accounted for almost two-thirds of the global new HIV infection in 2018 [18].

Several studies conducted previously showed differences in the amount of contraceptive utilization among HIV-positive reproductive-age women. The study done in Tigry zonal hospital (46.3%), Ethiopia [19] in the Amhara region (33.2%) [20], Addis Ababa (39.5%) [21], and Uganda (87.3%) [22] were reported. Education, awareness about contraception, occupation, family income, desire to have children, and a partner’s HIV status were the main factors associated with the pattern of contraceptive choice and utilization [10]. Despite intensive efforts to encourage condom use and improve access in sub-Saharan Africa, the amount of condom usage remains suboptimal [10]. There is a low acceptance of condom use in sub-Saharan Africa due to socio-cultural influences, gender and sexual norms, influences of poverty, and insufficient information [23]. Among the factors identified for low condom preference, refusal by the spouse or partner to use them [24].

Assessing the knowledge, attitude, and practice about family planning of women living with HIV helps identify the gaps that help stakeholders implement corrective action [25]. As there was no local study that assessed knowledge, attitude, and practice about family planning among women living with HIV, the authors designed this study. Therefore, the current study aimed to assess knowledge, attitude, and practice about family planning among women living with HIV at the University of Gondar comprehensive specialized hospital.

## Methods

### Study design, setting and period

An institution-based cross-sectional study was undertaken. The study was conducted in an ART clinic of the University of Gondar compressive and specialized hospital. The University of Gondar Hospital is one of the oldest academic institutions in Ethiopia. The University is situated at the heart of Gondar city, found in the Amhara Region, in the north-west part of Ethiopia, which is located 727 km away from Addis Ababa (the capital city of Ethiopia). The hospital provides different inpatient and outpatient services to the population in the surrounding area of Gondar town and the nearby zones [26]. In the University of Göndar hospital ART unit, around 5686 (5518 adults and 168 pediatrics) were registered in chronic care follow-up, of which about 1131 were female in the reproductive age groups at the time of the study period. The study was conducted from July 18 to August 18, 2022.

### Source and study population

The source populations were all women living with HIV and attending an ART follow-up clinic at the University of Gondar specialized hospital ART clinic. And the study populations were randomly selected HIV-positive women between the ages of 18 and 47 who got service from the University of Gondar specialized hospital ART clinic during the study period.

### Inclusion and exclusion criteria

In this study, women who live with HIV and are 18–47 years old, sexually active, and take ART medicine at least for six months were enrolled. These women who were diagnosed with cervical cancer, could not speak or hear, and were mentally ill were excluded.

### Sample size and sampling technique

The sample size (n) was calculated using a single population proportion formula by using the previous study from Eastern Ethiopia (good attitude = 35.7%) [27] and adding a non-response rate of 10%.

$$n = \frac{{{{\left( {{z_{\frac{a}{2}}}} \right)}^2} \times p \times \left( {1 - p} \right)}}{{{d^2}}} = \frac{{{{\left( {1.96} \right)}^2} \times 0.479 \times \left( {1 - 0.479} \right)}}{{{{\left( {0.05} \right)}^2}}}$$

By adding a 10% non-response rate, the final sample size was 387.

A systematic random sampling (SRS) technique was used by calculating the K value using the sampling frame. The sampling frame (list of women living with HIV and attending ART follow-up clinics) was obtained from the registration books of each follow-up clinic in the hospitals. The lottery method was used to select the first women.

### Study variables

#### Dependent variable:

knowledge, attitude, and practice of family planning.

#### Independent variables:

age, religion, residence, marital status, education status, occupation, monthly income, number of years since diagnosed, number of years since start of HAART, partner status on HIV, family size, number of children, desire of a child in the future, number of children living with HIV, number of births in the past 5 years, availability of contraceptive methods in their local area, source of information on family planning methods, purpose of family planning, partner interest in the same number of children as women, discussion about fertility plan with healthcare provider, and CD4 count.

### Data collection instrument and techniques

The data collection tool was a semi-structured, pretested interviewer-administered questionnaire to collect the required data adapted from the previous studies [10]. It had four parts. Part one included socio-demographic characteristics and related items of contraceptive use; part two included questions about knowledge of contraceptives; part three included items to assess attitudes toward contraceptive use; and part four included items to assess the practice of family planning. The electronic Kobo tool box was used for the data collection. Two graduating pharmacists were recruited for the data collection.

The principal researcher crafted all the items in English. Subsequently, a professional conducted the translation from English to Amharic. This forward translation was then reversed by a second professional into Amharic, revealing no disparities between the two English versions.

### Data quality control

A pretest was conducted among 15 participants out of the study area (Felegehiwot Comprehensive Specialized Hospital), and a few modifications were made. The data collectors were trained by the principal investigator for half a day about the purpose of the study, objectives, and ethical issues. The reliability test was done to check the internal consistency of the tool, and the values of Cronbach’s alpha test were 7.35 and 7.98 for attitude and practice, respectively.

### Data management and statistical analysis

The collected data were exported to Excel and then exported to SPSS Version 26 for computing, recording, and statistical analysis. The mean with standard deviation (SD) and frequency with percent were computed to represent the descriptive results of the study. Binary logistic regression was used to explain the relationship between the dependent variables (attitude and practice) and the independent variables. Independent variables with a *p*-value of < 0.2 were selected to be candidate variables for multiple logistic regression. Independent variables with a *p*-value of ≤ 0.05 with a 95% CI were declared as statistically significant factors for the outcome variables.

### Operational definition

#### Modern FP methods:

this refers to family planning methods such as pills, injectable (Depo-Provera), condoms, implants, and intrauterine contraceptive devices.

#### Dual method:

using condoms to prevent HIV transmission and also using at least one highly effective contraceptive method to prevent pregnancy.

#### Knowledge:

study participants were asked about safer conception, about the dual method, about modern contraceptive methods, and about several contrastive methods such as pill, implant, and injectable contraceptives. Those study participants who answered the questions correctly were considered to have good knowledge.

#### Attitude:

seven Likert items were asked and their results summed, and the mean (4.84) was used to classify participants (the data was normally distributed). Those study participants who scored above the mean were considered to have a good attitude.

#### Practice:

study participants were asked five serious questions and summed. The mean (2.72) was used to categorize good practice and poor practice (the data was normally distributed). Those study participants who scored above the mean were considered to have good practice.

## Results

### Socio demographic characteristics of the study participants

About three hundred and twenty-eight (328) women participated, with a response rate of 84.7%. The mean age with standard deviation (mean ± SD) of respondents was 35.2 ± 10.35 years. Among the study participants, 246 (75%) were orthodox Christians, 279 (85.9%) lived in urban areas, and 67 (20.4%) were illiterate. More than half of the study participants (54.6%) were currently married, and about half of the study participants (50.6%) had jobs. About half of the study participants (49.3%) have passed more than 7 years since they were diagnosed. 255 (77.7%) women have a CD4 count greater than 500. More than half (54%) of women reported that their partner was HIV-positive, and 41 (12.5%) women have an HIV-negative partner. About 60.7% of women have a desire to have a child in the future, and 90 (27.4%) participants have no child (Table 1).

**Table 1:** Socio-demographic characteristics and related variables among study participants, university of Gondar compressive specialized hospital, Gondar town, Ethiopia 2022 (*n* = 328)

Variable	Category	Frequency(n)	Percentage (%)
**Age**	18–27	77	23.5
	28–34	73	22.3
	35–40	101	30.8
	41–47	77	23.5
**Religion**	Orthodox	246	75
	Muslim	62	18.9
	Protestant	18	5.5
	Other	2	0.6
**Residence**	Urban	279	85.1
	Rural	49	17.6
**Marital status**	Married	179	54.6
	Unmarried	149	45.4
**Education status**	Illiterate	67	20.4
	Able to read and write	151	46.0
	College and above	110	33.5
**Occupation**	Have a job	166	50.6
	No job	162	49.4
**Monthly income (ETB Birr)**	0-1700	83	25.3
	1701–4000	97	29.6
	4001–5600	69	21.0
	5601–25,000	79	24.1
**Number of years since diagnosed**	1–7 years	165	50.3
	> 7 years	163	49.7
**Number of years since started ART**	1-7years	174	53.0
	> 7years	154	47
**Partner status on HIV**	Positive	180	54.9
	Negative	41	12.5
	Unknown	30	9.1
	No partner	77	23.5
**Family size**	1-3people	202	61.6
	>=4	126	38.4
**Number of children**	No child	90	27.4
	Have one or more child	238	72.6
**Desire of child in the future**	Yes	199	60.7
	No	129	39.3
**Number of children live with HIV**	0 child	244	74.6
	1 child	55	16.8
	>=2children	28	8.5
**Number of births in past 5 years**	0 (no birth)	184	56.1
	1 birth	118	36.0
	2 or more	26	7.9
**CD4 count**	< 500	73	22.3
	>=500	255	77.7

### Contraceptive use related information

About 59.2% of women got information about FP from a health care provider, and the majority (86.2%) of women have discussed fertility plans with healthcare providers. One hundred thirty-six (41.5%) women thought that family planning was used to prevent unwanted pregnancy, and 134 (40.9%) women thought that it was used to maintain a happy and healthy family. Two hundred and eighty-six (87.2%) women discussed their fertility plan with their health care provider. One hundred and seventy-five (53.4%) participants were pregnant after a confirmed HIV positive test, and of these, 151 (86.3%) women said it was planned. The result of pregnancy indicated that 127 (72.57%) were live-in birth, 26 (14.8%) were aborted, 9 (5.10%) were miscarried, 6 (3.42%) were still born, and 7 (4.06%) were pregnant at the time of study (Table 2).

**Table 2:** Contraceptive use and related information among study participants, university of Gondar compressive specialized hospital, Gondar town, Ethiopia 2022 (*n* = 328)

Variable	Category	Frequency	Percentage
Availability of contraceptive method in your local area	All are available	247	75.3(%)
	Some are available	58	17.7
	Not available	23	7.0
She sources of information on family planning methods	From family, friend and neighbor suggestion	32	9.8
	From health professional advice	227	59.2
	From media (TV, radio)	34	10.4
	I don’t know	35	10.7
For what purpose do you think family planning is used?	To prevent unwanted pregnancy	136	41.5
	To bring intentional pregnancy	19	5.8
	To maintain gap b/n 2 birth	39	11.9
	To maintain happy and healthy family	134	40.9
Does your partner want the same number of children as you?	Yes	190	57.9
	No	138	57.9
Having discussion on fertility plan with healthcare provider	Yes	286	87.2
	No	42	12.8
Ever been pregnant after HIV positive confirmed	Yes	175	53.4
	No	153	46.6
Paving pregnancy	Planned	151	86.3
	Unplanned	24	13.7
Result of pregnancy	Live birth	127	72.57
	Miscarriage	9	5.10
	Abortion	26	14.85
	Still birth	6	3.42
	Current pregnancy	7	4.06

### Respondents’ knowledge about family planning

**Fig. 1:**
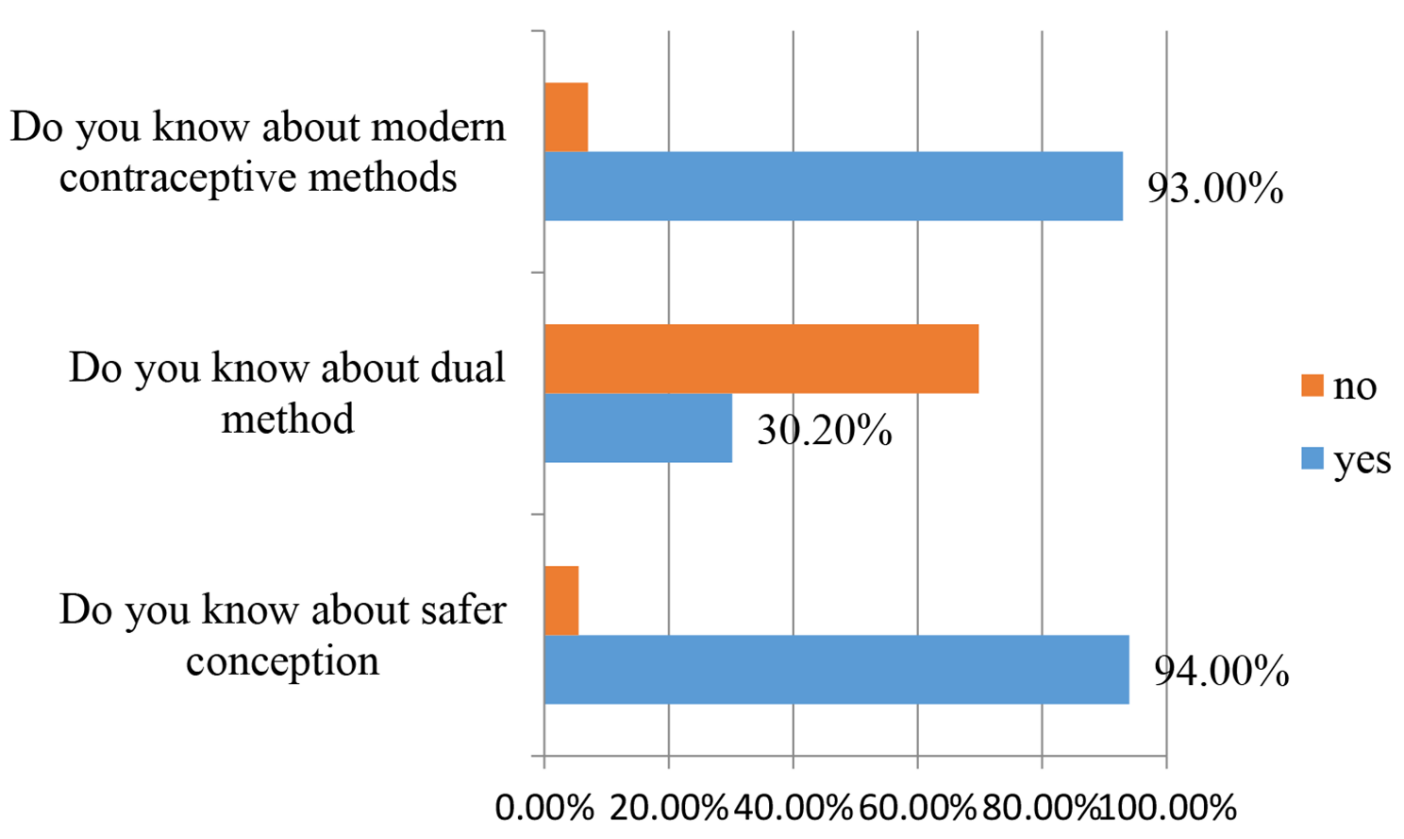
Knowledge of study participants on family planning (*n* = 328)

**Fig. 2:**
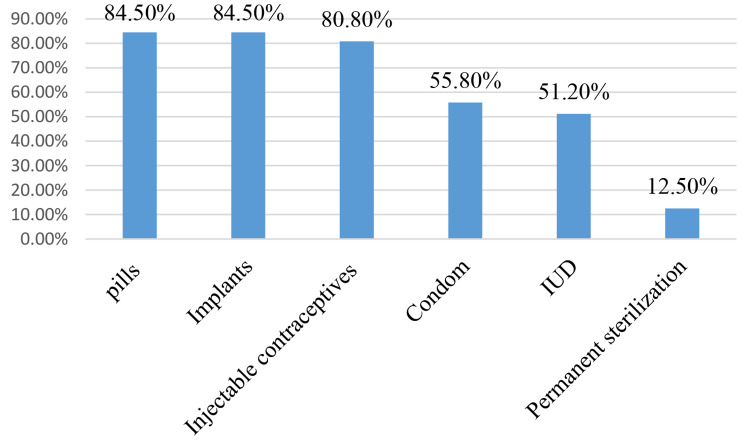
Knowledge of study participants on modern contraceptive methods (*n* = 328)

Three hundred and ten (94.5%) women had good knowledge about safer conception; 99 (30.5%) women had good knowledge about the dual method; and 305 (93.0%) women had good knowledge about modern contraceptive methods (Fig. 1). About 84.5% of the study participants had good knowledge of pills and implants, about 80.8% of the study participants had good knowledge of injectable contraceptives, and about 55.8% of the study participants had good knowledge of condoms (Fig. 2).

### Attitudes and practice of study participants towards family planning

**Fig. 3:**
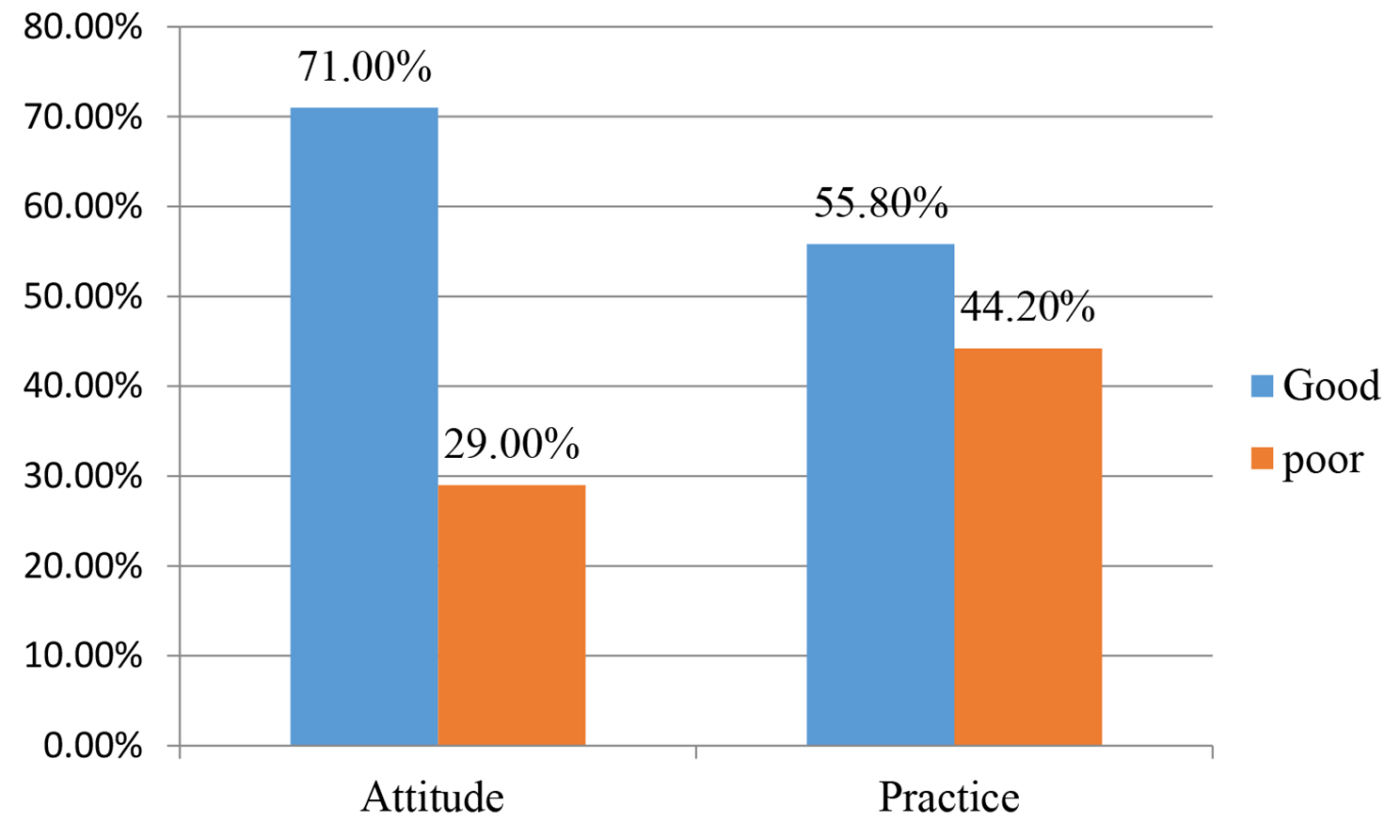
Attitude and practice on family planning among the study participants (*n* = 328)

**Fig. 4:**
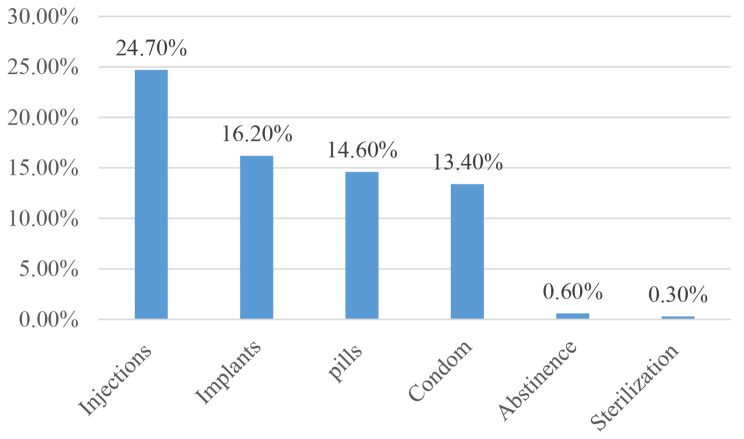
Utilization of contraceptives among the study participants (*n* = 328)

In the current study, 71.0% (95% CI: 66.5–75.9%) participants had a good attitude, and 55.8% (95% CI: 50.3–61.0%) participants had good practice (Fig. 3). The most commonly used contraceptives in the current study were inactions (24.7%), followed by implants (16.1%), pills (14.6%), and condoms (13.4%) (Fig. 4).

### Reasons for abstain of family planning practice

In the current study, one hundred and seventy-seven (54.0%) participants did not use contraceptives, and from those participants, 57 (32.0%) women were due to needing birth less than 2 years, 11 (6.21%) women were due to one of them being infertile, 97 (54.8%) women were due to not having a partner or sex, and 12 (6.97%) women were due to fear of side effects. Two hundred and eighty-five (86.9%) participants did not use the dual method because they didn’t know where to get them (40.50%), they didn’t know how to use them (46.83%), and their partner refused them 36 (12.67%) (Table 3).

**Table 3:** Reasons for abstain of family planning practice of study participants (*n* = 328)

Variable	Category	Frequency	Percentage
**Do you and your partner use contraceptive now**	Yes	151	46.0
	No	177	54.0
**Reasons for not to use contraception**	Desire birth in less than 2 years	57	32.20
	One of us is infertile	11	6.21
	No sex/partner	97	54.80
	Fear of side effect	12	6.79
**Do you use dual method to prevent transmission**	Yes	43	13.1
	No	285	86.9
**Reasons not to use dual method**	Do not know where to get them	115	40.50
	Do not know how to use them	133	46.83
	Partner refuse to use them	36	12.67

### Associated factors of attitude on family planning

In the current study, age, family size, number of children, occupation, education status, marital status, having discussions on fertility plans with healthcare providers, knowledge about modern contraceptive methods, and FP practice were candidate variables for multiple logistic regression (*p* < 0.2). In the final model, having one and a greater number of children had about 2.25 times (AOR = 2.25, 95% CI: 1.10, 4.49) a better attitude than women who have no child. Study participants who had discussions on fertility plans with healthcare providers had a 2.2-times (AOR = 2.20, 95% CI: 1.02, 4.761) better attitude than their counterparts. Study participants with good practice of family planning had 2.15 times higher (AOR = 2.15, 95% CI: 1.19, 3.87) than study participants with poor family planning practice (Table 4).

**Table 4:** Associated factors of good attitude towards family planning among women with HIV (*n* = 328)

Variable	Category	Attitude		COR	AOR
		Positive n	Negative n		
Age	18–27	55	22	1	1
	28–34	52	21	0.51(0.23,1.10)	0.99(0.37,2.630
	35–40	62	39	0.50(0.23,1.10)	0.47(0.19,1.16)
	41–47	64	13	0.32(0.16,0.66)	0.28(0.12,0.62)
Family size	1–3	131	71	2.30(1.36,3.91)	1.54(0.79,3.01)
	>=4	102	24	1	1
Number of children	No children	52	38	1	1
	Have one or more children	181	57	2.32(1.39,3.88	2.25(1.10,4.59)*
Occupation	Have a job	125	41	1.52(0.94,2.47)	1.12(0.60,2.09)
	No job	108	54	1	1
Education status	Illiterate	45	22	1	1
	Able to read and write	98	53	0.90(0.49,1.66)	0.82(0.40,1.67)
	Collage and above	90	20	2.20(1.09,4.45)	1.24(0.49,3.12)
Marital status	Married	134	45	1.50(0.93,2.43)	1.14(0.64,2.02)
	Unmarried	99	50	1	1
having discussion on fertility plan with healthcare provider	Yes	213	73	3.21(1.66,6.21)	2.20(1.02,4.761)*
	No	20	22)	1	1
Know about modern contraceptive methods	Yes	224	81	4.30(1.79,10.32)	2.12(0.77,5.86)
	No	9	14	1	1
FP practice	Poor practice	84	61	1	1
	Good practice	149	34	3.18(1.94,5.23)	2.15(1.19,3.87)*

Hosmer and Lemeshow Test = 0.74 * ***p*** **< 0.05**

### Associated factors of practice on family planning

In the current study, marital states, educational status, occupation, years since confirmed HIV positive, family size, having discussions on fertility plans with healthcare providers, knowledge about safer conception, knowledge about dual methods, and knowledge about modern contraceptive methods were candidate variables for multiple logistic regression (*p* < 0.2). In the final model, married women had about 1.88 times (AOR = 1.88, 95% CI = 1.11–3.1) better family planning practices than unmarried women. Education status was significantly associated with family planning practice, as indicated by those women who were able to read and write having about 2.12 times (AOR = 2.12, 95% CI: 1.04–4.32) better family planning practice than illiterate women, and those women with college and above had 4.51 times (AOR = 4.51, 95% CI: 1.93–10.87) better family planning practice than illiterate women. Study participants who had discussions on fertility plans with healthcare providers had 5.09 times (AOR = 5.09, 95% CI: 1.96, 13.24) better family planning practices than those study participants who did not have discussions on fertility plans with healthcare providers. Study participants who knew about the dual method had 1.95 times (AOR = 1.95, CI: 1.08, 3.50) better practice of family planning than their counterparts, and study participants who knew about modern contraceptive methods had 7.24 times (AOR = 7.24, 95% CI: 1.56, 33.58) better practice of family planning than their counterparts (Table 5).

**Table 5:** Associated factors of good practice on family planning of women live with HIV (*n* = 328)

Variable	Category	Practice		COR	AOR
		Good n	Poor n		
Marital states	Married	121	58	2.93(1.86,4.60)	1.88(1.11,3.17)*
	Unmarried	62	87	1	1
Educational status	illiterate	27	40	1	1
	Able to read and write	46	75	1.50(0.84,2.69)	2.12(1.04,4.32)*
	Collage and above	80	30	3.95(2.08,7.52)	4.57(1.93,10.87*)
Occupation	Have a job	104	62	1.76(1.13,2.73)	1.48(0.80,2.72)
	No job	79	83	1	1
Years since confirmed HIV positive	1- 7 years	103	62	1.72(1.11,2.67)	1.75(0.98,3.06)
	>7years	80	83	1	1
Family size	1–3	99	103	2.08(1.31,3.30)	1.34(0.72,2.50)
	>=4	84	42	1	1
Having discussion on fertility plan with healthcare provider	No	8	34	1	1
	Yes	175	111	6.70(2.99,15.00)	5.09(1.96,13.24)*
know about safer conception	No	7	11	1	1
	Yes	176	134	2.06(0.78,5.47)	1.73(0.50,5.97)
Know about dual method	No	114	115	1	1
	Yes	69	30	2.32(1.41,3.83)	1.95(1.08,3.50)*
Know about modern contraceptive methods	No	2	21	1	1
	Yes	181	124	15.33(3.33,65.55)	7.24(1.56,33.58)*

Hosmer and Lemeshow Test = 0.69 * ***p*** **< 0.05**

## Discussion

The current study assessed the knowledge, attitude, and practice of FP among women living with HIV at the University of Gondar Specialized Hospital. In the current study, the majority of the study participants had a high level of awareness of safer conception (94.5%) and modern contraceptive methods (93.0%). This is almost similar to a previous study conducted in Ethiopia, in which 95.4% of study participants had awareness of modern FP methods [28]. The results also align with previous research conducted in Tanzania, which reported a high level of knowledge about contraception methods among the participants [29]. This suggests that women living with HIV in different regions share a similar level of knowledge regarding family planning options. The result is also somewhat higher than the previous studies conducted in Uganda (74.1%) [30] and Nigeria (64.8%) [31]. The difference in methods, study setting, healthcare system, and sociocultural methods might be attributed to the possible variation.

The study showed that the majority (71.0%) of study participants had a good attitude towards family planning. The finding is similar to a study conducted in Nigeria, with a high proportion of participants exhibiting positive attitudes towards family planning [32]. In the current study, more than half (55.8%) of the study participants had good practice in family planning. The most commonly utilized contraceptives in this study were injections (24.7%), followed by implants (16.1%), pills (14.6%), and condoms (13.4%). These findings differ from studies conducted in multiple African countries that found that condoms were the most commonly used contraceptive method among women living with HIV [33], and are lower than the study done in Nepal [34]. These variations may be attributed to differences in healthcare infrastructure, the availability of contraceptive methods, and cultural norms regarding contraceptive use.

The reasons for abstaining from family planning were the desire to give birth within a short interval or concerns about infertility. This finding is consistent with findings from other studies [35–38]. This suggests that these factors serve as prevalent obstacles to family planning among women with HIV in various settings.

The associated factors with attitude and practice of family planning were identified in this study using logistic regression. Having one or a greater number of children, having discussions on fertility plans with healthcare providers, and having a good practice of family planning were significantly associated with a good attitude toward family planning in the current study. Married women, education status, discussions on fertility plans with healthcare providers, knowledge about dual methods, and knowledge about modern contraceptive methods were significantly associated with better family planning practices.

Study participants who had good practice in family planning had a good attitude towards the family planning method. This is supported by the previous study [39]. It highlights the role of practice in influencing women’s perceptions of family planning methods. When individuals actively incorporate family planning into their lives, they may experience firsthand the benefits in terms of better control over family size, spacing of pregnancies, and overall reproductive health. This positive experience can contribute to a more favorable attitude towards family planning methods.

Having discussions on fertility plans with healthcare providers was significantly associated with good family planning practices. This is supported by the previous studies [38–41]. This indicated the potential benefits of seeking guidance from healthcare providers in making informed, personalized, and comfortable decisions regarding family planning. Previous research may have indicated that personalized guidance from healthcare providers contributes to better adherence to family planning practices [42].

Married study participants had good family planning practices similar to the previous study [40]. This might be due to discussions about how contraceptive issues with a partner contribute to good family planning practices, which are proposed based on the understanding that open communication fosters informed decision-making and mutual support [41].

Having discussions with healthcare providers and knowledge about modern contraceptive methods has also been reported in previous research conducted in Kenya, which found that women who had discussions about family planning with healthcare providers were more likely to have positive attitudes and higher contraceptive use [43]. Similarly, a study conducted in Eastern Ethiopia [39] and China among women living with HIV reported that knowledge about modern contraceptive methods was associated with higher contraceptive use [44]. These findings reinforce the importance of healthcare provider counseling and comprehensive knowledge in promoting positive attitudes and practices toward family planning among this population.

Education status was significantly associated with family planning practice, as indicated by the fact that those women who were able to read and write had about 2.12 times better family planning practice than illiterate women, and those women with college and above had 4.51 times better family planning practice than illiterate women. This study is consistent with studies done in eastern Ethiopia [38] and Malawi [45] that showed education status has a strong association with good family planning practices. This is not surprising, as education status is a multifaceted factor influencing family planning practices through increased access to information, enhanced awareness, improved decision-making skills, empowerment, socioeconomic status, cultural changes, and a better understanding of contraceptive methods.

Even though the results of this study provide valuable insights into the specific context of this population, it has certain limitations. The study relied on self-reported data, which introduced the possibility of social desirability bias. Participants may provide answers that they believe are more socially acceptable, leading to a potential overestimation or underestimation of certain behaviors or attitudes. The study may not fully capture the influence of social and cultural factors on family planning practices among women living with HIV. These factors play a significant role in shaping attitudes, decision-making, and access to contraception and should be considered in future research.

## Conclusion and recommendations

This study highlighted the relatively high levels of knowledge about modern contraceptive methods and safer conception among women living with HIV. However, knowledge of dual methods was low. More than two-thirds of the study population had a good attitude about family planning. More than half of the study population had good practice in family planning. Having one or a greater number of children, having a discussion on a fertility plan with a healthcare provider, and having a good practice of family planning were significantly associated with a good attitude toward family planning. Married women, education status, discussions on fertility plans with healthcare providers, knowledge about dual methods, and knowledge about modern contraceptive methods were significantly associated with good family planning practices. Therefore, healthcare providers should prioritize comprehensive counseling and open discussions about family planning with women living with HIV to address the aforementioned associated factors of attitude and practice in family planning. Conducting further research with larger sample sizes and diverse settings to better understand the factors influencing family planning practices among women living with HIV would be advisable.

## Data Availability

The data is available at the correspondence author upon reasonable request.

## Abbreviations

AIDS: acquired immune deficiency syndrome
ART: anti-retroviral therapy
FP: family planning
HIV: human immune deficiency syndrome
IUD: intrauterine device
OCS: oral contraceptives
PLHIV: people who live with HIV
PMCT: prevention of mother-to-child transmission
SCS: safer conception strategies
WHIV: women with HIV
